# The Establishment of Complement System Is from Gene Duplication and Domain Shuffling

**DOI:** 10.3390/ijms25158119

**Published:** 2024-07-25

**Authors:** Jiejie Sun, Chang Liu, Lingling Wang, Linsheng Song

**Affiliations:** 1 Liaoning Key Laboratory of Marine Animal Immunology, Dalian Ocean University, Dalian 116023, China; sunjiejie@dlou.edu.cn (J.S.); liuchang@dlou.edu.cn (C.L.); wanglingling@dlou.edu.cn (L.W.); 2 Liaoning Key Laboratory of Marine Animal Immunology & Disease Control, Dalian Ocean University, Dalian 116023, China; 3 Dalian Key Laboratory of Aquatic Animal Diseases Prevention and Control, Dalian Ocean University, Dalian 116023, China

**Keywords:** complement system, metazoan, evolution, terminal complement component

## Abstract

The mammalian complement system constitutes a highly sophisticated body defense machinery. The evolutionary origin of the complement system can be traced to Coelenterata as the presence of the central component C3 and two activation proteases BF and MASP. In the present study, the main complement components were screened and analyzed from the genomes of different species in metazoan subphyla/phyla. C1q with classical domains can be traced to Annelida, and ficolin and MBL to Urochordata. C1r and C1s are only found in Chondrichthyes and even higher species, and MASP is traced to Coelenterata. In the evolutionary tree, C1r from Vertebrates is close to MASP1/2/3 from Deuterostomia and Coelenterata, and C1s from Vertebrates is close to MASP-like protease (MASPL) from Arthropoda, Mollusca, and Annelida. C2, BF, and DF can be traced to Mollusca, Coelenterata, and Porifera, respectively. There are no clear C2 and BF branches in the evolutionary tree. C3 can be traced to Coelenterata, and C4 and C5 are only in Chondrichthyes and even higher species. There are three clear C3, C4, and C5 branches in the evolutionary tree. C6-like (C6L) and C8 can be traced to Urochordata, and C7-like (C7L) can be traced to Cephalochordara. C6L, C7L, and C8 from Urochordata and Cephalochordara provide the structural conditions for the formation of Vertebrate MAC components. The findings unveil the evolutionary principles of the complement system and provide insight into its sophistication.

## 1. Introduction

The complement system, as one of the most sophisticated innate immune systems, consists of three independent but interacting pathways [1,2]. It plays crucial roles in recognizing and destroying pathogenic microorganisms as well as in eliminating modified self-antigens [3]. Recently, as molecular and bioinformatics research proceeds, the evolutionary origin of the complement system was revealed to be increasingly ancient [1,4,5,6]. Hence, it is necessary to understand a wider range of animal phylogeny to follow the evolutionary process of the complement system.

The mammalian complement system is composed of more than 30 components present mainly in serum and cell membranes and plays essential roles in innate immunity [2,7]. Once activated, a chain reaction of proteolysis and assembly of protein complexes evolves, which is finely regulated by soluble and membrane-bound regulators [8]. Complement activation can be initiated through any of its three activation pathways, classical, alternative, or lectin, all converging toward the formation of C3-convertases and cleavage of the C3 component into anaphylatoxins C3a and C3b. C3b is involved in the formation of C5-convertase, which in turns cleaves C5 into anaphylatoxins C5a and C5b. C5b interacts with C6, C7, C8, and several C9 proteins to form the membrane attack complex (C5b-9 or MAC), which generates a lytic pore in the target membrane [9,10].

The complement system in mammals is composed of three independent pathways (classical, lectin, and alternative pathways) and a terminal complement pathway [1,2]. The origin and evolution of the complement system have been studied in metazoans [8,11]. In the classical pathway, C1q with a collagen-like domain has now been traced to bivalves [12], and C1r, C1s, C2, and C4 have been identified in bony fishes [13,14,15]. In the lectin pathway, MBL with a collagen-like domain has been identified in lamprey [16] and ascidians [17,18]. And ficolin, with a collagen-like domain, is found in mammals, birds, reptiles, amphibians, amphioxus [19], and ascidians [20]. MASP can be traced to Coelenterata [1]. In the alternative pathway, BF is present in Coelenterata and DF is only reported in mammals. Apart from this, the key component, C3, is also found in the genome of Coelenterata [1,21]. By contrast, no complement gene was found in the genomes of *Drosophila melanogaster* [22] and *Caenorhabditis elegans* [23]. The genes possessing exactly the same or very similar structural domains as human terminal complement component (TCC) genes have been identified from all classes of extant jawed vertebrates, including Teleosts [1,24] and Chondrichthyes [25].

The accumulation of genomic information of many representative animals has made it possible to trace the evolution of the complement system based on the presence or absence of each complement component in the analyzed genomes. In the present study, the presence and structural and evolutionary characteristics of complement components are systematically analyzed according to the genomes of species in major metazoan subphyla/phyla with the objectives to clarify and deduce the origin and evolution of complement components and even the complement system in metazoans.

## 2. Results

### 2.1. The Distribution, Structural Domain, and Phylogenetic Tree Analysis of C1qs, Ficolins, and MBLs

**Figure 1:**
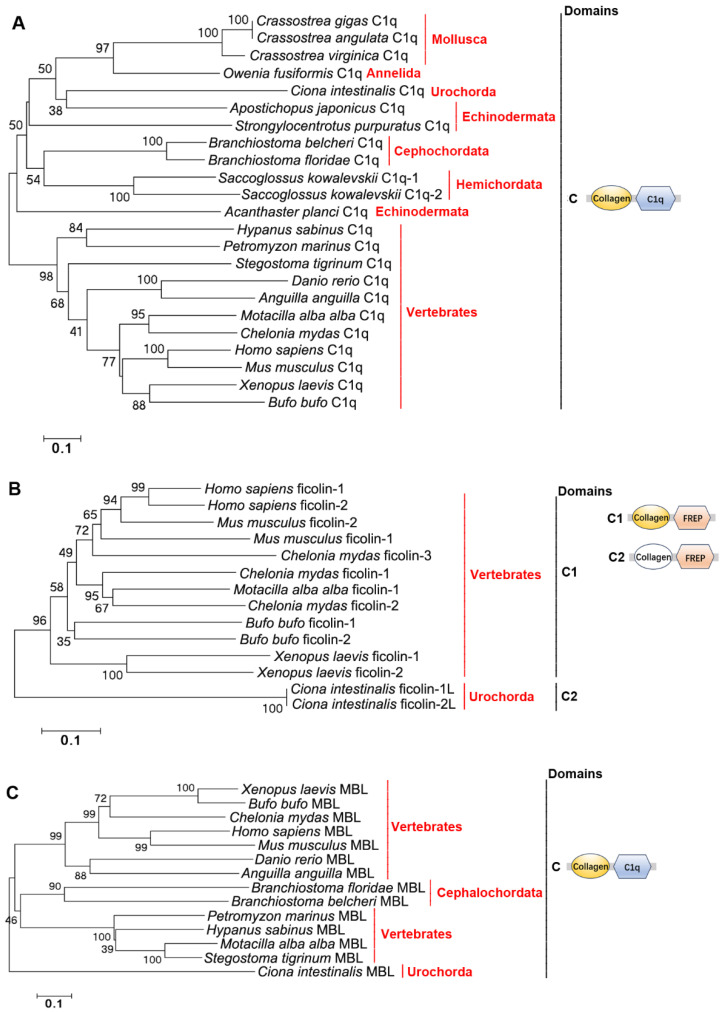
The evolutionary analysis and structural domains of C1qs, ficolins, and MBLs in different species of metazoans. (**A**) The evolutionary analysis and structural domains of C1qs. *H. sapiens* C1q, NP_001334395.1; *M. musculus* C1q, NP_031598.2; *M. alba alba* C1q, XP_038015420.1; *X. laevis* C1q, XP_018097336.1; *B. bufo* C1q, XP_040279956.1; *C. mydas* C1q, EMP42096.1; *D. rerio* C1q, AJP77497.1; *A. anguilla* C1q, XP_035289015.1; *H. sabinus* C1q, XP_059807651.1; *S. tigrinum* C1q, XP_048417810.2; *P. marinus* C1q, XP_032834306.1; *B. belcheri* C1q, KAI8500951.1; *B. floridae* C1q, XP_035695925.1; *C. intestinalis* C1q, XP_018667470.1; *A. japonicus* C1q, AOR82889.1; *S. purpuratus* C1q, XP_003724148.1; *A. planci* C1q, XP_022088206.1; *S. kowalevskii* C1q-1, XP_002739445.1; *S. kowalevskii* C1q-2, XP_002732902.2; *O. fusiformis* C1q, CAH1795369.1; *C. gigas* C1q, XP_011416082.1; *C. virginica* C1q, XP_022318030.1; *C. angulata* C1q, XP_052688239.1. (**B**) The evolutionary analysis and structural domains of ficolins. The collagen domain marked with white indicates the primitive domain. *H. sapiens* ficolin-1, NP_001994.2; *H. sapiens* ficolin 2, KAI4009060.1; *M. musculus* ficolin-1, NP_032021.1; *M. alba alba* ficolin-1, XP_038011850.1; *X. laevis* ficolin-1, XP_041421702.1; *X. laevis* ficolin-2, XP_018124342.2; *B. bufo* ficolin-1, XP_040298222.1; *B. bufo* ficolin-2, XP_040264661.1; *C. mydas* ficolin-1, XP_007067693.2; *C. mydas* ficolin-2, XP_043386624.1; *C. mydas* ficolin-3, XP_007064883.2; *C. intestinalis* ficolin-1L, XP_009859735.2; *C. intestinalis* ficolin-2L, XP_018668937.1. (**C**) The evolutionary analysis and structural domains of MBLs. *H. sapiens* MBL, AAK52907.1; *M. musculus* MBL, NP_001351987.1; *M. alba alba* MBL, XP_037989255.1; *X. laevis*, MBL, XP_041424875.1; *B. bufo* MBL, XP_040293381.1; *C. mydas* MBL, XP_043407079.1; *D. rerio* MBL, NP_001108197.1; *A. anguilla* MBL, XP_035280239.1; *H. sabinus* MBL, XP_059813153.1; *S. tigrinum* MBL, XP_048387692.2; *P. marinus* MBL, XP_032810447.1; *B. floridae* MBL, P_035672354.1; *B. belcheri* MBL, XP_019613692.1; *C. intestinalis* MBL, XP_026692805.1. Bootstrap values lower than 30 were omitted from the tree. The domain names are given in Table 1. C: Classical structural feature.

C1qs, ficolins, and MBLs containing a classical collagen domain were screened from genomes of well-annotated species in four metazoan subphyla (Vertebrates, Cephochordata, Urochordata, and Hemichordata) and seven phyla (Echinodermata, Brachiopoda, Arthropoda, Mollusca, Annelida, Coelenterata, and Porifera). C1qs are present in Vertebrates, Cephochordata, Urochordata, Hemichordata, Echinodermata, Mollusca, and Annelida, while they are lost in Brachiopoda, Arthropoda, Coelenterata, and Porifera (Figure 1A). Ficolins are found in Vertebrates and Urochordata (Figure 1B). MBLs are in Vertebrates, Cephalochordata, and Urochordata (Figure 1C). C1qs, ficolins, and MBLs all contain a collagen domain. In addition, they also have C1q, FREP, and CRD domains, respectively (Figure 1A–C). The screened C1qs, ficolins, and MBLs were employed to construct the evolutionary tree using the Neighbor-Joining (NJ) method, respectively. Among them, there are two branches for C1qs. C1qs from Vertebrates are clustered together into one branch. The other C1qs are dropped into another branch (Figure 1A). Ficolins from Vertebrates are clustered together and ficolin-like receptors (ficolinLs) from Urochordata are clustered together as the sister group of ficolins from Vertebrates (Figure 1B). MBLs are divided into three branches. MBLs from Teleosts and even higher species are clustered together as one branch. MBLs from Cephochordata are close to those from Cyclostomata and Chondrichthyes, and they are clustered together as a second branch. MBL from Urochordata is in a single branch (Figure 1C).

### 2.2. The Distribution, Structural Domain, and Phylogenetic Tree Analysis of C1rs, C1ss, and MASPs

**Figure 2:**
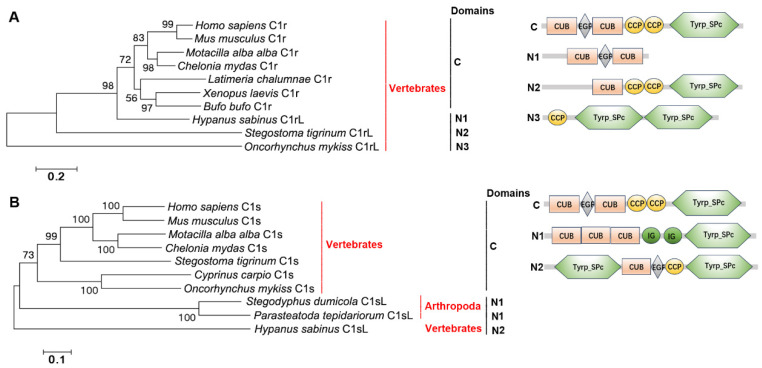
The evolutionary analysis and structural domains of C1rs and C1ss in different species of metazoans. (**A**) The evolutionary analysis and structural domains of C1rs. *H. sapiens* C1r, AAA51851.1; *M. musculus* C1r, NP_075632.3; *M. alba alba* C1r, XP_038004514.1; *X. laevis* C1r, NP_001090130.1; *B. bufo* C1r, XP_040294837.1; *C. mydas* C1r, XP_027690298.1; *O. mykiss* C1r, XP_036797195.1; *L. chalumnae* C1r, XP_006013027.1; *H. sabinus* C1r, XP_059815579.1; *S. tigrinum* C1r, XP_059500004.1. (**B**) The evolutionary analysis and structural domains of C1ss. *H. sapiens* C1s, KAI2564157.1; *M. musculus* C1s, NP_776289.2; *M. alba alba* C1s, XP_038004527.1; *C. mydas* C1s, XP_007072391.1; *H. sabinus* C1s, XP_059832311.1; *C. carpio* C1s, XP_042597174.1; *O. mykiss* C1s, XP_021437654.2; *S. tigrinum* C1s, XP_048378174.1; *S. dumicola* C1s, XP_035220366.1; *P. tepidariorum* C1s, XP_042898361.1. Bootstrap values lower than 30 were omitted from the tree. The domain names are given in Table 1. C: Classical structural feature; N: Nonclassical structural feature.

**Figure 3:**
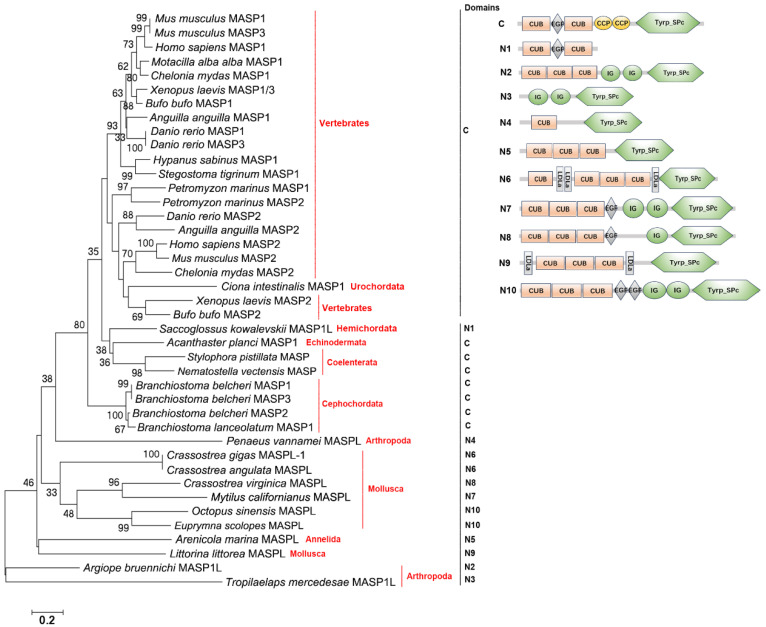
The evolutionary analysis and architectures of MASPs in different species of metazoans. *H. sapiens*, MASP1, NP_624302.1; *H. sapiens* MASP2, EAW71674.1; *M. musculus* MASP1, NP_001346012.1; *M. musculus* MASP2, NP_001003893.1; *M. musculus* MASP3, BAB69688.1; *M. alba alba* MASP1, XP_038002309.1; *X. laevis* MASP1/3, AAH73178.1; *X. laevis* MASP2, BAA86865.1; *C. mydas* MASP1, XP_037764893.1; *C. mydas* MASP2, EMP41528.1; *B. bufo* MASP1, XP_040284327.1; *B. bufo* MASP2, XP_040286182.1; *D. rerio* MASP1, NP_001373185.1; *D. rerio* MASP2, NP_001116330.1; *D. rerio* MASP3, CDU85283.1; *A. anguilla* MASP1, XP_035242085; *A. anguilla* MASP2, XP_035236672.1; *H. sabinus* MASP1, XP_059846853.1; *S. tigrinum* MASP1, XP_059507155.1; *P. marinus* MASP1, XP_032817860.1; *P. marinus* MASP2, XP_032818062.1; *B. belcheri* MASP1, BAC75888.1; *B. belcheri* MASP2, XP_019637434.1; *B. belcheri* MASP3, BAC75889.1; *B. lanceolatum* MASP1, CAH1240236.1; *C. intestinalis* MASP1, XP_018672886.1; *S. kowalevskii* MASP1L, XP_006821215.1; *A. planci* MASP1, XP_022109261.1; *A. bruennichi* MASP1L, XP_055949985.1; *T. mercedesae* MASP1L, OQR79970.1; *P. vannamei* MASPL, ROT74645.1; *A. marina* MASP2L, UCK81482.1; *C. gigas* MASPL-1, XP_011447092.3; *C. virginica* MASPL, XP_022302791.1; *C. angulata* MASPL, XP_052689041.1; *L. littorea* MASPL, QBA18416.1; *O. sinensis* MASPL, *M. californianus* MASPL, XP_052067819.1; *E. scolopes* MASPL, AFV94411.1; *S. pistillata* MASP, XP_022800644.1; *N. vectensis* MASP, XP_032226040.1. Bootstrap values lower than 30 were omitted from the tree. The domain names are given in Table 1. C: Classical structural feature; N: Nonclassical structural feature.

C1rs, C1ss, and MASPs were screened from genomes of well-annotated species in four metazoan subphyla and seven phyla. C1rs are present in Vertebrates and C1ss are in Vertebrates and Arthropoda (Figure 2A,B). MASPs are widely present in Vertebrates, Cephochordata, Urochordata, Hemichordata, Echinodermata, Arthropoda, Mollusca, Annelida, and Coelenterata (Figure 2). C1rs from Amphibia and even higher Vertebrates have the classical CUB-EGF-CUB-CCP_2_-Tyrp_SPc domain. C1r-like proteases (C1rLs) from Chondrichthyes and Teleosts have partial domains, such as the CUB-EGF-CUB domain of C1rL from *Hypanus sabinus*, the CUB-CCP_2_-Tyrp_SPc domain of C1rL from *Stegostoma tigrinum*, and the CCP-Tyrp_SPc_2_ domain of C1rL from *Oncorhynchus mykiss*. C1ss from Teleosts and even higher Vertebrates have the classical CUB-EGF-CUB-CCP_2_-Tyrp_SPc domain. C1s-like protease (C1sL) from Chondrichthyes has the Tyrp_SPc-CUB-EGF-CCP-Tyrp_SPc domain, and C1sL from Arthropoda has the CUB-EGF-CUB-IG_2_-Tyrp_SPc domain, lacking the two CCP domains. MASPs from Vertebrates, Cephochordata, Urochordata, Echinodermata, and Coelenterata have the classical CUB-EGF-CUB-CCP_2_-Tyrp_SPc domain. MASP-like proteases (MASPL) from other phyla all lack the two CCP domains, some of which are replaced by two IG domains (Figure 2). In the two evolutionary trees of C1rs and C1ss, those with the classical domains are clustered together, respectively (Figure 2A,B). In the evolutionary trees of MASPs, those from Vertebrates and Urochordata are clustered together (Figure 3). MASPs from Cephochordata, Hemichordata, Echinodermata, and Coelenterata are clustered together as the sister group of those from Vertebrates and Urochordata. In addition, MASPLs from Arthropoda, Mollusca, and Annelida are clustered together (Figure 3).

**Figure 4:**
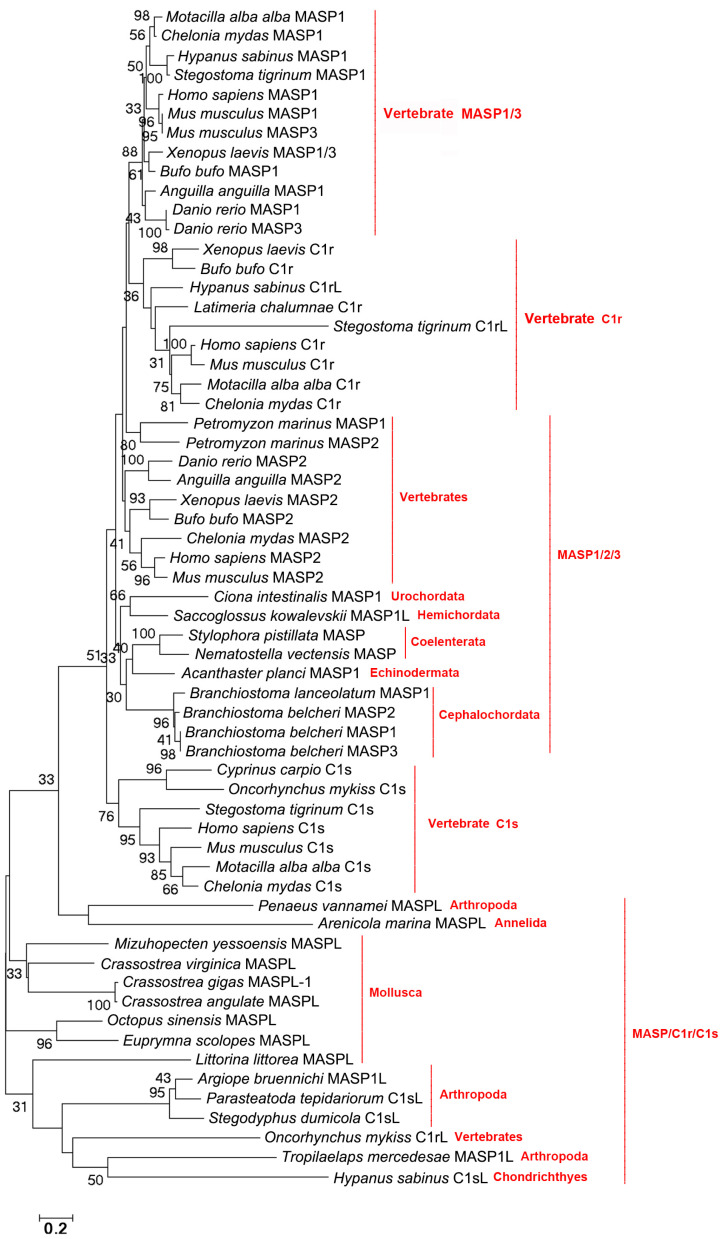
The evolutionary analysis of C1rs, C1ss, and MASPs in different species of metazoans. Bootstrap values lower than 30 were omitted from the tree.

The evolutionary tree was constructed using the amino acid sequences of C1rs, C1ss, and MASPs to further analyze their relationships. MASP1/3s from Vertebrates are clustered together, and C1rs and MASP2s from Vertebrates are clustered together as a sister group of MASP1/3 from Vertebrates (Figure 4). MASPs from Cephalochordata, Urochordata, Hemichordata, Echinodermata, and Coelenterata are clustered together and they are close to Vertebrate MASP2s. C1s from Vertebrates are clustered together and they are close to MASPLs from Arthropoda, Mollusca, and Annelida (Figure 4).

### 2.3. The Distribution, Structural Domain, and Phylogenetic Tree Analysis of C2s, BFs, and DFs

**Figure 5:**
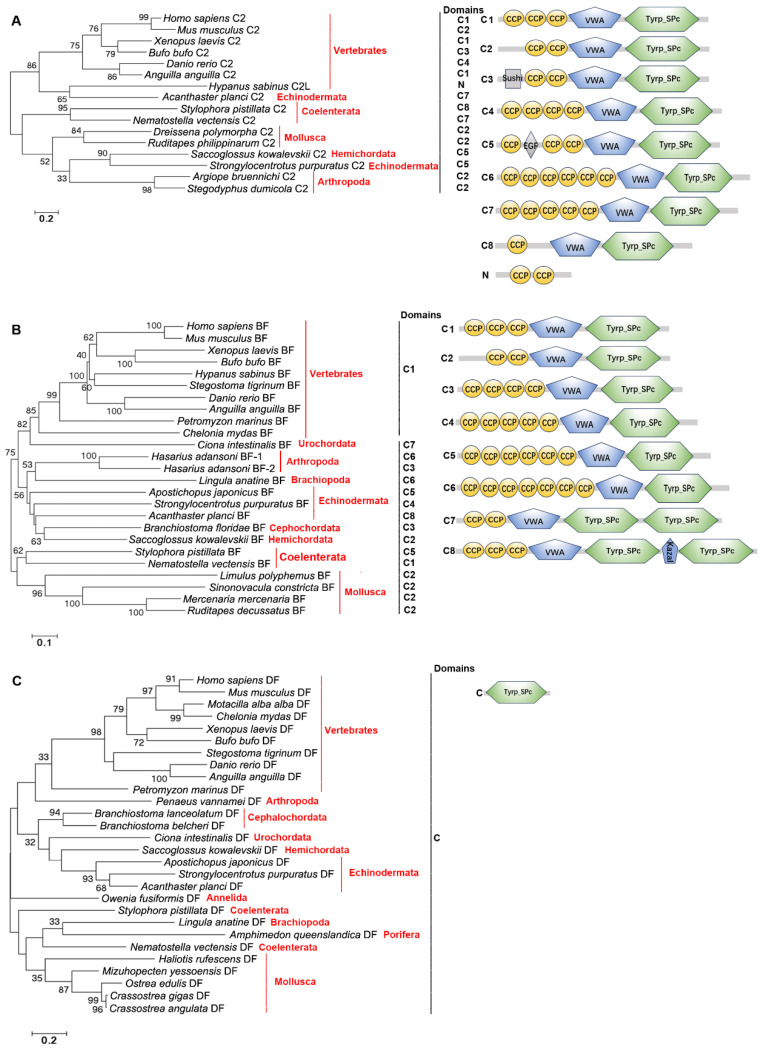
The evolutionary analysis and structural domains of C2s, BFs, and DFs in different species of metazoans. (**A**) The evolutionary analysis and structural domains of C2s. *H. sapiens* C2, UQL51015.1; *M. musculus* C2, NP_038512.2; *X. laevis* C2, NP_001116166.2; *B. bufo* C2, XP_040261399.1; *D. rerio* C2, XP_009289626.1; *A. anguilla* C2, XP_035239999.1; *H. sabinus* C2, XP_059809249.1; *S. kowalevskii* C2, XP_006826065.1; *S. purpuratus* C2, XP_030853781.1; *A. planci* C2, XP_022099169.1; *A. bruennichi* C2, XP_055939093.1; *S. dumicola* C2, XP_035216049.1; *D. polymorpha* C2, XP_052224032.1; *R. philippinarum* C2, XP_060586248.1; *S. pistillata* C2, XP_022806518.1; *N. vectensis* C2, XP_032240896.2. (**B**) The evolutionary analysis and architectures of BFs. *H. sapiens* BF, CAA51389.1; *M. musculus* BF, NP_032224.2; *X. laevis* BF, AAI69984.1; *B. bufo* BF, XP_040262351.1; *C. mydas* BF, XP_037754704.1; *D. rerio* BF, XP_021324426.1; *A. anguilla* BF, XP_035287799.1; *H. sabinus* BF, XP_059827115.1; *S. tigrinum* BF, XP_048376055.2; *P. marinus* BF, XP_032818095.1; *C. intestinalis* BF, NP_001027973.1; *B. floridae* BF, XP_035685199.1; *S. kowalevskii* BF, XP_006820863.1; *A. japonicus* BF, ADX36428.1; *S. purpuratus* BF, NP_999700.1; *A. planci* BF, XP_022108946.1; *H. adansoni* BF-1, BAR45590.1; *H. adansoni* BF-2, BAR45591.1; *L. anatine* BF, XP_013415956.1; *M. mercenaria* BF, XP_045197948.2; *R. decussatus* BF, ACQ91095.1; *S. constricta* BF, QEX93860.1; *L. polyphemus* BF, XP_013783723.2; *S. pistillata* BF, XP_022808245.1; *N. vectensis* BF, BAH22728.1. (**C**) The evolutionary analysis and structural domains of DFs. *H. sapiens* DF, KAI2587505.1; *M. musculus* DF, NP_038487.1; *M. alba alba* DF, XP_038019812.1; *X. laevis* DF, NP_001088093.1; *B. bufo* DF, XP_040273465.1; *C. mydas* DF, XP_027690140.1; *D. rerio* DF, NP_001018368.1; *A. anguilla* DF, XP_035268129.1; *S. tigrinum* DF, XP_048395876.1; *P. marinus* DF, XP_032810884.1; *B. lanceolatum* DF, CAH1255086.1; *B. belcheri* DF, XP_019638506.1; *C. intestinalis* DF, XP_002126930.1; *S. kowalevskii* DF, XP_006813580.1; *A. japonicus* DF, PIK62543.1; *S. purpuratus* DF, XP_030844513.1; *A. planci* DF, XP_022088740.1; *L. anatine* DF, XP_013420482.1; *P. vannamei* DF, XP_027226181.1; *O. fusiformis* DF, CAH1794826.1; *C. gigas* DF, XP_034313728.1; *C. angulata* DF, XP_052689168.1; *O. edulis* DF, XP_056020373.1; *M. yessoensis* DF, OWF41022.1; *H. rufescens* DF, XP_048248666.1; *S. pistillata* DF, PFX24724.1; *N. vectensis* DF, EDO47761.1; *A. queenslandica* DF, XP_019864501.1. Bootstrap values lower than 30 were omitted from the tree. The domain names are given in Table 1. C: Classical structural feature; N: Nonclassical structural feature.

C2s, BFs, and DFs were screened from the same genomes of the species mentioned above. C2s, BFs, and DFs are present in Vertebrates, Hemichordata, Echinodermata, Arthropoda, Mollusca, and Coelenterata. In addition, BFs are also in Cephalochordata, Urochordata, and Brachiopoda. DFs are in Cephalochordata, Urochordata, Brachiopoda, Annelida, and Porifera (Figure 5A–C). C2s and BFs all have the CCP_1–7_-VWA-Tyrp_SPc_2_ domains, except for C2 in Chondrichthyes, which only has two CCP domains (Figure 5A,B). DFs all have a classical Tyrp_SPc domain (Figure 5C). In the three evolutionary trees of C2s, BFs, and DFs, those from Vertebrates are clustered together, respectively. C2 from Echinodermata, BF from Urochordata, and DF from Arthropoda are close to those from Vertebrates, respectively. C2s, BFs, and DFs from other remaining species are clustered together, respectively (Figure 5A–C).

**Figure 6:**
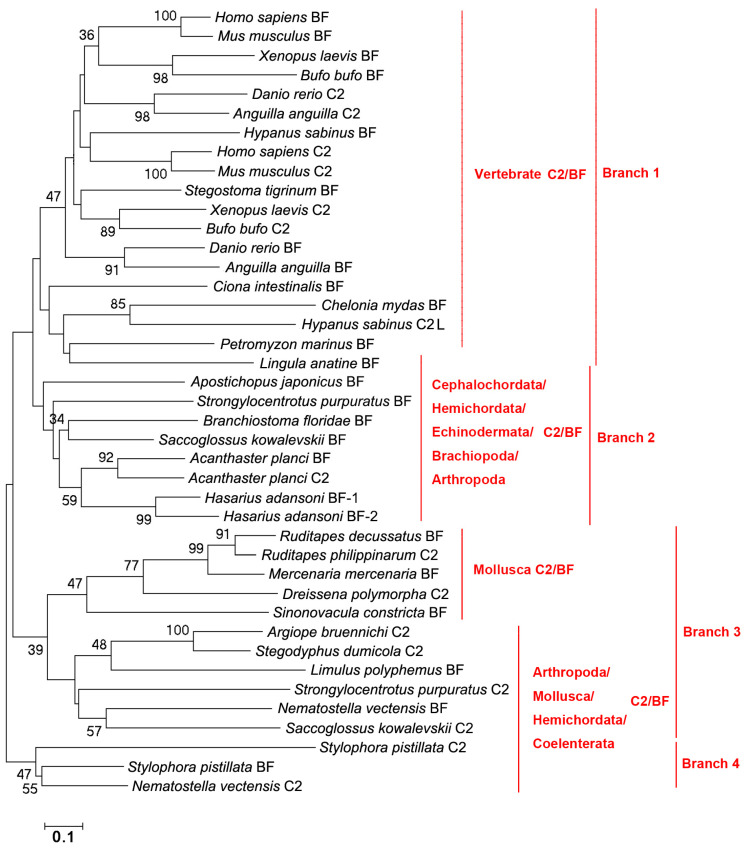
The evolutionary analysis of C2s and BFs in different species of metazoans. Bootstrap values lower than 30 were omitted from the tree. The domain names are given in Table 1.

The evolutionary tree was constructed using the amino acid sequences of C2s and BFs to further analyze their relationships. No clear C2 branch or BF branch is observed in the evolutionary tree. There are four branches, and C2s and BFs from Vertebrates are clustered together. BF from Branchiopoda is dropped into the branch of C2s and BFs from Vertebrates (Figure 6). C2s and BFs from Cephalochordata, Hemichordata, Echinodermata, Brachiopoda, and Arthropoda are in the second branch and they are close to those from Vertebrates. C2s and BFs from remaining species of Hemichordata, Arthropoda, Mollusca, and Coelenterata are in the third and fourth branches (Figure 6).

### 2.4. The Distribution, Structural Domain, and Phylogenetic Tree Analysis of C3s, C4s, and C5s

**Figure 7:**
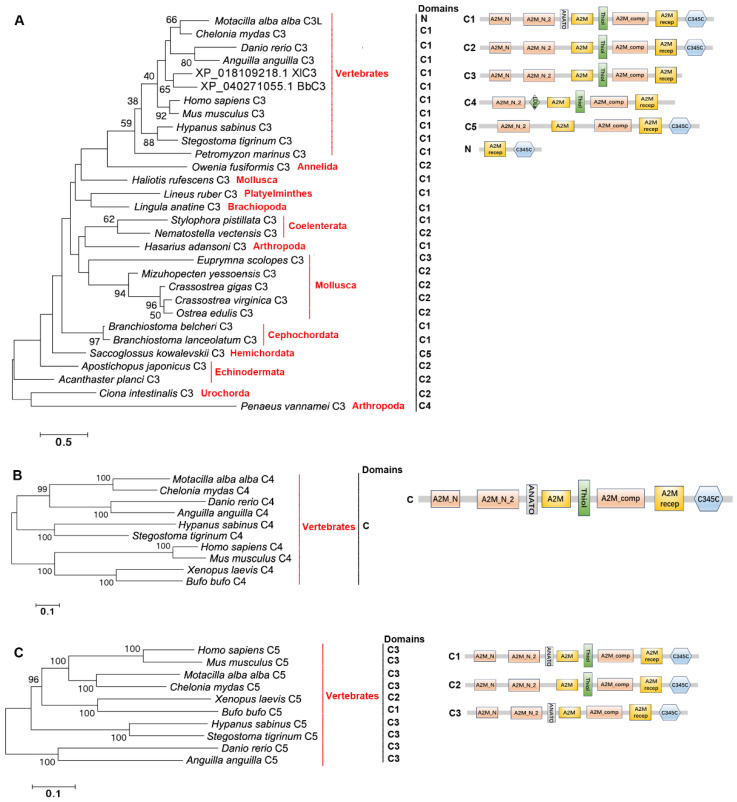
The evolutionary analysis and structural domains of C3s, C4s, and C5s in different species of metazoans. (**A**) The evolutionary analysis and structural domains of C3s. *H. sapiens* C3, NP_000055.2; *M. musculus* C3, AAH43338.1; *M. alba alba* C3, XP_037982332.1; *X. laevis* C3, XP_018109218.1; *B. bufo* C3, XP_040271055.1; *C. mydas* C3, XP_037743983.1; *D. rerio* C3, XP_009293521.1; *A. anguilla* C3, XP_035260736.1; *H. sabinus* C3, XP_059848367.1; *S. tigrinum* C3, XP_048377825.1; *P. marinus* C3, XP_032809867.1; *C. intestinalis* C3, NP_001027684.1; *B. belcheri* C3, BAB47146.1; *B. lanceolatum* C3, CAH1241049.1; *S. kowalevskii* C3, XP_006823058.1; *A. japonicus* C3, ADN97000.1; *A. planci* C3, XP_022108952.1; *O. fusiformis* C3, CAH1797988.1; *L. anatine* C3, XP_013420340.1; *P. vannamei* C3, XP_027223519.1; *H. adansoni* C3, BAK64110.1; *L. ruber* C3, WBU98461.1; *C. gigas* C3, XP_034335787.1; *C. virginica* C3, XP_022345651.1; *O. edulis* C3, XP_048730861.2; *M. yessoensis* C3, OWF37722.1; *H. rufescens* C3, XP_048245024.1; *E. scolopes* C3, ACF04700.1; *S. pistillata* C3, XP_022788977.1; *N. vectensis* C3, XP_048586922.1. (**B**) The evolutionary analysis and structural domains of C4s. *H. sapiens* C4, AAA51855.1; *M. musculus* C4, AAA39506.1; *M. alba alba* C4, XP_037988603.1; *X. laevis* C4, AAI70422.1; *B. bufo* C4, XP_040260692.1; *C. mydas* C4, XP_043401598.1; *D. rerio* C4, XP_005157429.1; *A. anguilla* C4, XP_035241768.1; *H. sabinus* C4, XP_059826670.1; *S. tigrinum* C4, XP_048419811.2. (C) The evolutionary analysis and structural domains of C5s. *H. sapiens* C5, AAA51925.1; *M. musculus* C5, XP_017171158.1; *M. alba alba* C5, XP_038012535.1; *X. laevis* C5, XP_041428294.1; *B. bufo* C5, XP_040261943.1; *C. mydas* C5, XP_007061045.3; *D. rerio* C5, XP_001919226.4; *A. anguilla* C5, XP_035234662.1; *H. sabinus* C5, XP_059849800.1; *S. tigrinum* C5, XP_059494269.1. Bootstrap values lower than 30 were omitted from the tree. The domain names are given in Table 1. C: Classical structural feature; N: Nonclassical structural feature.

**Figure 8:**
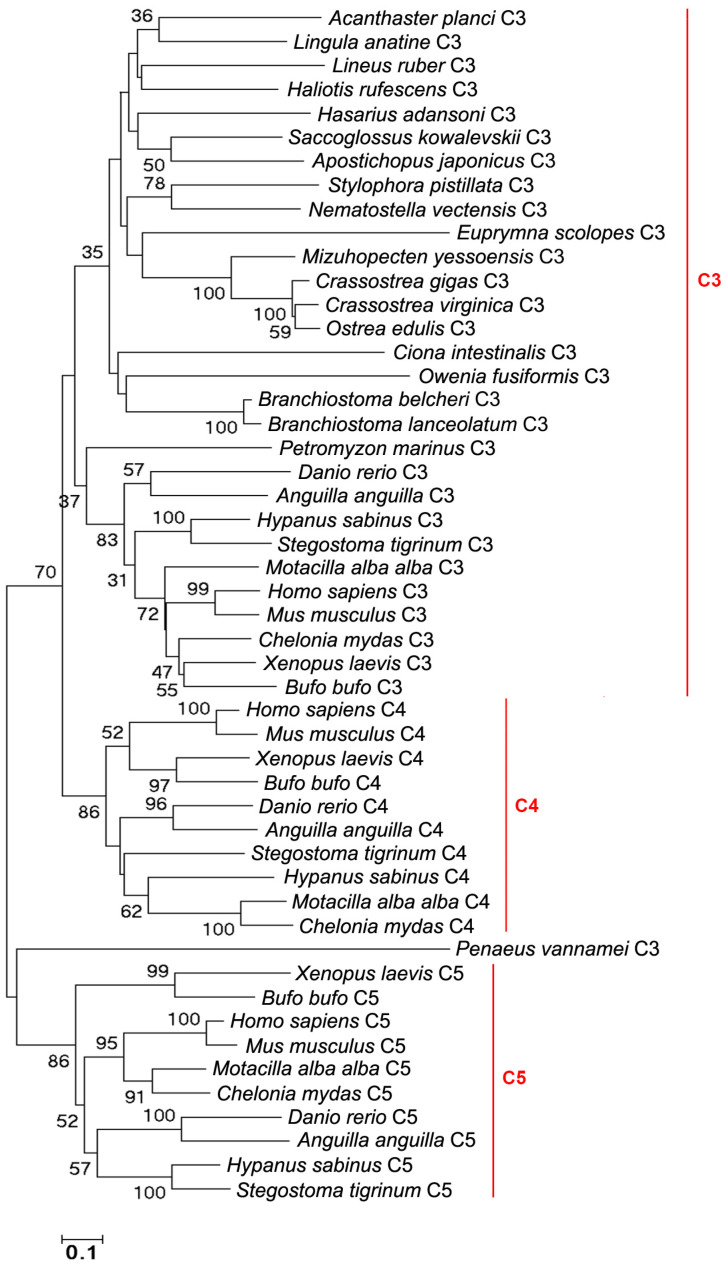
The evolutionary analysis of C3s, C4s, and C5s in different species of metazoans. Bootstrap values lower than 30 were omitted from the tree.

C3s, C4s, and C5s were screened from the same genomes of the species mentioned above. C3s are present in Vertebrates, Cephalochordata, Urochordata, Hemichordata, Echinodermata, Brachiopoda, Arthropoda, Mollusca, Annelida, Platyelminthes, and Coelenterata (Figure 7A). C4s and C5s are only in Vertebrates (Figure 7B,C). C3s from Vertebrates (except for *Motacilla alba alba*), Cephalochordata, Urochordata, Echinodermata, Brachiopoda, Arthropoda (except for *Penaeus vannamei*), Mollusca (except for *Euprymna scolopes*), Annelida, Platyhelminthes, and Coelenterata have the classical A2M_N-A2M_N_2-A2M-Thiol-A2M_comp-A2MR-C345C domain. C3s from *M. alba alba*, *S. kowalevskii*, *P. vannamei* and *E. scolopes* have the A2MR-C345C domain, A2M_N_2-A2M-A2M_comp-A2MR-C345C domain, A2M_N_2-LDLa-A2M-Thiol-A2M_comp-A2MR domain, and A2M_N-A2M_N_2-A2M-Thiol-A2M_comp-A2MR domain, respectively (Figure 7A). C4s and C5s all have the classical A2M_N-A2M_N_2-A2M-A2M_comp-A2MR-C345C domain (Figure 7B,C). C3s, C4s, and C5s from Vertebrates are clustered together, respectively. C3s from the remaining species are clustered together (Figure 7A). The evolutionary tree was constructed using the amino acid sequences of C3s, C4s and C5s to further analyze their evolution relationships. There are clear three branches (C3 branch, C4 branch, and C5 branch) in the evolutionary tree (Figure 8).

### 2.5. The Distribution, Structural Domain, and Phylogenetic Tree Analysis of C6s, C7s, C8s, and C9s

**Figure 9:**
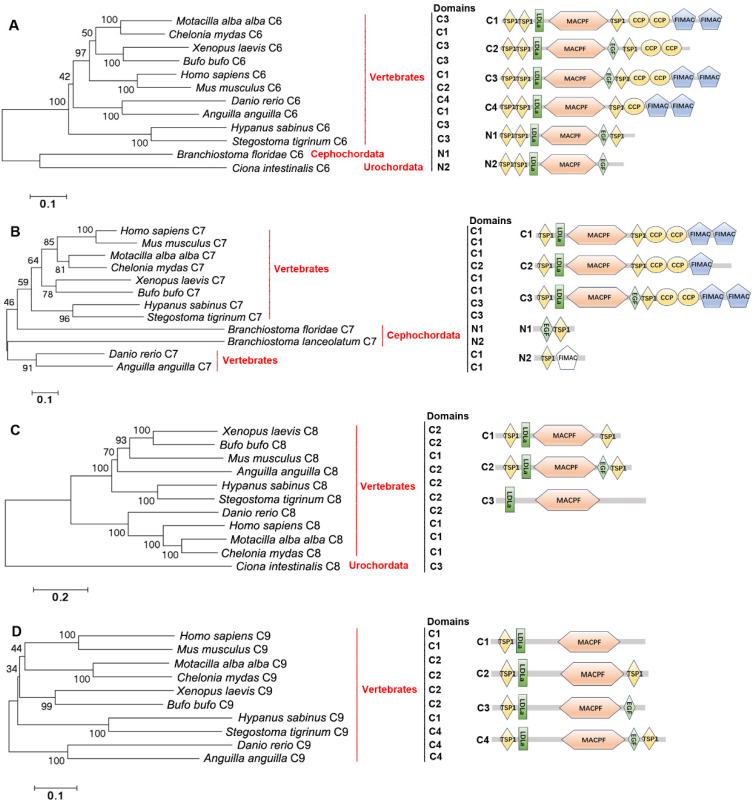
The evolutionary analysis and structural domains of C6s, C7s, C8s, and C9s in different species of metazoans. (**A**) The evolutionary analysis and structural domains of C6s. *H. sapiens* C6, NP_001108603.2; *M. musculus* C6, XP_006520020.1; *M. alba alba* C6, XP_037980997.1; *X. laevis* C6, NP_001079439.1; *B. bufo* C6, XP_040276861.1; *C. mydas* C6, XP_007065783.2; *D. rerio* C6, XP_005161216.1; *A. anguilla* C6, XP_035245908.1; *H. sabinus* C6, XP_059830244.1; *S. tigrinum* C6, XP_059500945.1; *B. floridae* C6, XP_035675448.1; *C. intestinalis* C6, XP_002130679.1. (**B**) The evolutionary analysis and structural domains of C7s. *H. sapiens* C7, AAA51861.1; *M. musculus* C7, NP_001230766.1; *M. alba alba* C7, XP_037981037.1; *X. laevis* C7, NP_001085116.1; *B. bufo* C7, XP_040276863.1; *C. mydas* C7, XP_043403285.1; *D. rerio* C7, XP_009302346.1; *A. anguilla* C7, XP_035291383.1; *H. sabinus* C7, XP_059830247.1; *S. tigrinum* C7, XP_059500946.1; *B. floridae* C7, XP_035685719.1; *B. lanceolatum* C7, CAH1232856.1. The FIMAC domain marked with white indicates the primitive domain. (**C**) The evolutionary analysis and structural domains of C8s. *H. sapiens* C8, EAX06641.1; *M. musculus* C8, BAC41363.1; *M. alba alba* C8, XP_038000853.1; *X. laevis* C8, NP_001085549.1; *B. bufo* C8, XP_040263170.1; *C. mydas* C8, XP_007059126.2; *D. rerio* C8, NP_001243652.1; *A. anguilla* C8, XP_035280356.1; *H. sabinus* C8, XP_059839579.1; *S. tigrinum* C8, XP_048395589.1; *C. intestinalis* C8, XP_009860510.2. (**D**) The evolutionary analysis and structural domains of C9s. *H. sapiens* C9, AAH20721.1; *M. musculus* C9, AAH11137.1; *M. alba alba* C9, XP_037980238.1; *X. laevis* C9, XP_018118713.1; *B. bufo* C9, XP_040276874.1; *C. mydas* C9, XP_037758636.1; *D. rerio* C9, NP_001314855.1; *A. anguilla* C9, XP_035245900.1; *H. sabinus* C9, XP_059830232.1; *S. tigrinum* C9, XP_059501030.1. Bootstrap values lower than 30 were omitted from the tree. The domain names are given in Table 1. C: Classical structural feature; N: Nonclassical structural feature.

C6s, C7s, C8s, and C9s were screened from the same genomes of the species mentioned above. They are present in Vertebrates (except for Cyclostomata). Besides, C6-like (C6L) is also present in Cephalochordata and Urochordata (Figure 9A). C7-like (C7L) is in Cephalochordata and C8 is in Urochordata (Figure 9B,C). C9 is not found in Cephalochordata and Urochordata (Figure 9D). C6s from Vertebrates (except for *Mus musculus*) all have the TSP1_2_-LDLa-MACPF-EGF_0–1_-TSP1-CCP_1–2_-FIMAC_2_ domain. C6 from *M. musculus* lacks the two FIMAC domains (Figure 9A). C6Ls from Cephalochordata and Urochordata have the TSP1_2_-LDLa-MACPF-EGF-TSP1_0–1_ domain (Figure 9A). C7s from Vertebrates all have the TSP1-LDLa-MACPF-EGF_0–1_-TSP1-CCP_2_-FIMAC_1–2_ domain. C7L from Cephalochordata only has the EGF-TSP1 domain or TSP1-FIMAC domain (Figure 9B). C8s and C9s from Vertebrates all have the classical TSP1_0–1_-LDLa-MACPF-EGF_0–1_-TSP1_0–1_ domain (Figure 9C,D). Also, C8 from Urochordata has the LDLa-MACPF domain (Figure 9C). C6s, C7s, C8s, and C9s from Chondrichthyes are close to those of Amphibia and even higher animals, respectively, compared with those from Teleosts (Figure 9A–D). C6s and C8s from Vertebrates are clustered together (Figure 9A,C). C7Ls from *Branchiostoma floridae* and *B. lanceolatum* of Cephalochordata are clustered together with C7s of Chondrichthyes and Teleosts, respectively (Figure 9B).

**Figure 10:**
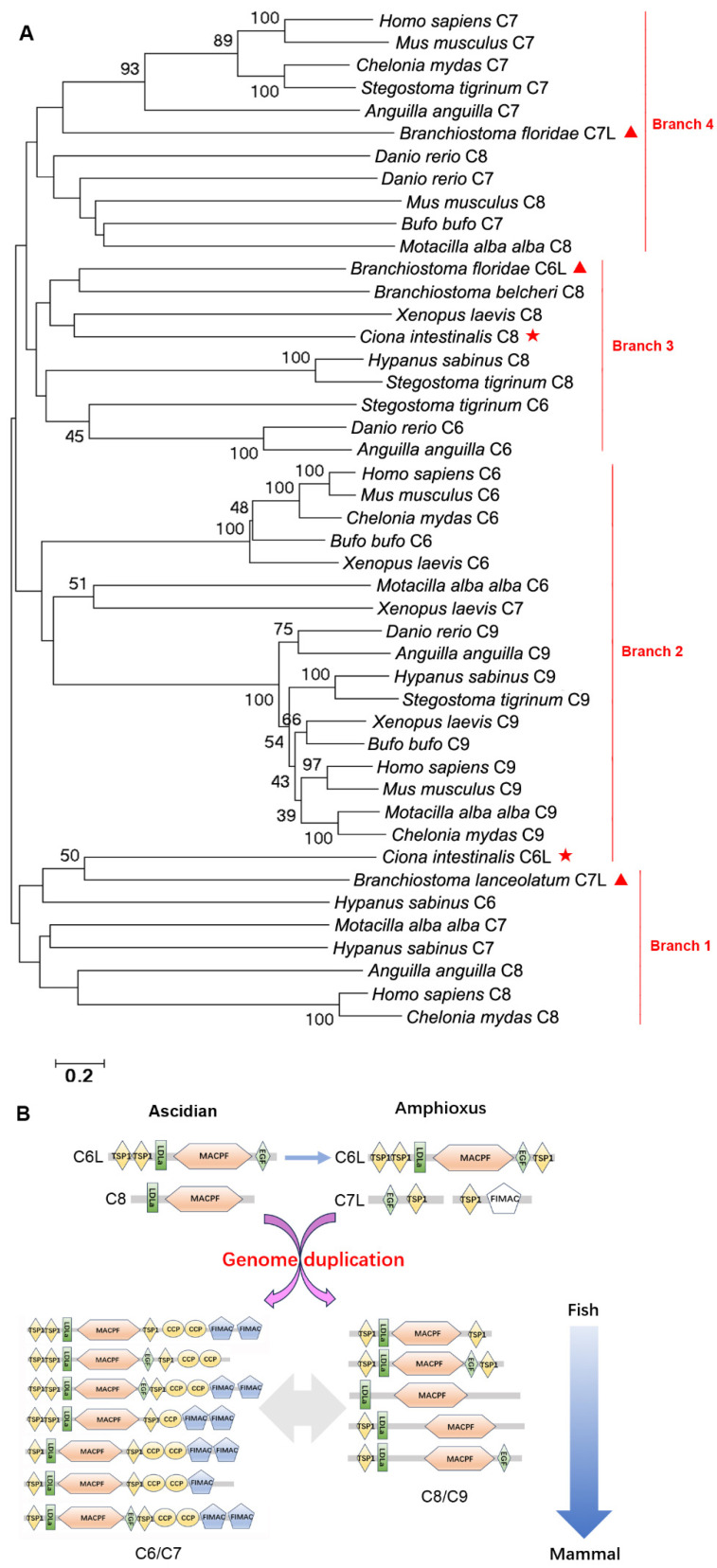
The evolutionary analysis of C6s, C7s, C8s, and C9s and a schematic of their origin. (**A**) The evolutionary analysis of C6s, C7s, C8s, and C9s. Bootstrap values lower than 30 were omitted from the tree. The domain names are given in Table 1. (**B**) A schematic of the origin of C6s, C7s, C8s, and C9s. The MAC components in vertebrates originate from C6L, C7L, and C8 in Ascidian and Amphioxus. The genes from Ascidian marked with red asterisk and those from Amphioxus marked with red triangle.

In the evolutionary tree of C6s, C7s, C8s, and C9s, there are four clear branches (Figure 10A). C6, C7s, and C8s from some Vertebrates are clustered together with C6L from Urochordata and C7L from Cephalochordata in one branch. Most C6s and all C9s from Vertebrates are in the second branch. C6s and C8s from fish are clustered together with C8 from Urochordata and C6L from Cephalochordata in the third branch. Most C7s and some C8s from Vertebrates are clustered together with C7L from Cephalochordata in the fourth branch (Figure 10A).

To investigate the origin of MAC, a comprehensive analysis of the domain architectures of MAC components was conducted (Figure 10B). C6L from Amphioxus evolves from C6L from Ascidian. C6L and C8 from Ascidian and C7L from Amphioxus provide the structural conditions for Vertebrate MAC components. Among them, C6L and C8 offer the TSP1, MACPF, and EGF domains, and C7L provides the EGF, TSP1, and primitive FIMAC domains. These structural foundations along with replication of the fish genome eventually form C6, C7, C8, and C9 containing classical TSP1, MACPF, EGF, and/or FIMAC (Figure 10B).

## 3. Discussion

The mammalian complement system is composed of more than 30 components present mainly in serum and cell membranes and plays essential roles in innate immunity [1,26,27]. The complement system is activated through three pathways: the classical pathway, the lectin pathway, and the alternative pathway. The classical pathway is initiated through binding to C1q. The lectin pathway is initiated through association with ficolin or MBL. The alternative pathway is constitutively activated through a so-called “tickover” mechanism [28]. Activation of the complement system leads to the cleavage of C3. The generated C3b can form a covalent bond with C3b or C4b of C3 convertases, switching their specificity to C5 convertases [29]. Proteolytic activation of C5 initiates the assembly of late components, C6 to C9, leading to the formation of MACs, which disturbs the integrity of the cell membranes of microbes [30]. In the present study, the origin and evolution of the complement system were studied by systematically analyzing the complement components possessing these unique structural domains in metazoan subphyla/phyla.

The classical and lectin pathways can be activated through binding to the first components, C1q and ficolin/MBL, respectively [31]. Among them, classical pathway activation occurs after binding of C1q to antibody–antigen complexes, cell particles, or certain acute phase proteins, such as C-reactive protein or serum amyloid P. The lectin pathway is activated when ficolin/MBL interacts with carbohydrate structures present on invading pathogens [32]. C1q and ficolin/MBL form hexamers through their collagen domain to recognize their corresponding ligands [33]. In the present study, the classical recognition molecules such as C1q, ficolin, and MBL all have the classical collagen domain. The classical C1qs can be traced to Annelida, and the classical ficolins and MBLs can be traced to Urochordata [17,20]. Meanwhile ficolins with collagen domains are lost in Cephochordata, Cyclostomata, Chondrichthyes, and Teleosts. Ficolins and MBLs lacking the collagen domain widely exist in different invertebrate phyla [34,35,36]. In Cephochordata, *Bj*FCN1 from *B. japonicum* lacking the collagen domain was also demonstrated to interact with MASP1/3 to induce the activation of C3 [19], indicating that ficolins lacking the collagen domain also could activate the lectin pathway. Also, in Mollusca, MBL-like protein lacking the collagen domain also could activate the lectin pathway [37]. These results further illustrate that although C1q, ficolin, and MBL in lower invertebrates lack the collagen domain, they may also have the function to activate the complement system. In addition, although in Urochordata there are primitive collagen domain-containing ficolin and MBL, their functions in activating the complement system are still not clear [20,38].

The classical and lectin pathways are initiated by C1r/C1s (classical) and MASP-1/MASP-2/MASP-3 (lectin) proteases [28]. The classical C1r/C1 contains the CUB-EGF-CUB-CCP-CCP-Tyrp_SPc domain. In the present study, C1rs and C1ss with classical domains are traced to Amphibia and Teleosts, respectively. And in even lower fishes, the domains of C1rs and C1ss are not conserved. C1rL in Teleosts has the CCP-Tyrp_SPc-Tyrp_SPc domain and in Chondrichthyes, C1rL has the CUB-EGF-CUB domain or CUB-CCP-CCP-Tyrp_SPc domain, suggesting that the classical structural domains of C1r are formed by the fusion of the CUB-EGF-CUB domain and CUB-CCP-Tyrp_SPc domain in C1rL from Chondrichthyes. The results also indicate that C1rL in Chondrichthyes has the most primitive structural feature. C1sL in Chondrichthyes has the Tyrp_SPc-CUB-EGF-CCP-Tyrp_SPc domain, which is also the most primitive structural feature of C1s. Although in Arthropoda shrimp there is a C1s homolog, its two CCP domains are replaced by two IG domains. The classical MASP is composed of the classical CUB-EGF-CUB-CCP-CCP-Tyrp_SPc domain. In the present study, MASP with classical domains is traced back to Coelenterata. But it is lost in Mollusca and even higher species. Until the appearance of Echinodermata, MASPs with classical domains reappear. MAPSLs in Arthropoda, Mollusca, and Annelida lose the two CCP domains and some have the additional two IG domains. Also, the domain arrangement of MASPs in Arthropoda, Mollusca, and Annelida is quite different from those with the classical domains. These results also indicate that there is a great change in the structural domains of MASPLs in Annelida, Mollusca, Arthropoda, and Hemichordata. In Mollusca, although MASP lacks the two CCP domains, it could also induce the cleavage of C3 [37], indicating that although the two CCP domains of C1r/C1s and MASP homologs from most lower invertebrates were replaced by two IG domains, they also have the same function as that of classic C1r/C1s and MASP. Although the domains of C1r, C1s, and MASP differ greatly in different phyla of metazoans, their structures are similar in the same phyla. The evolutionary tree was constructed to further analyze their relationships with each other. MASP1/3s from Vertebrates are clustered together, and C1rs and MASP2s from Vertebrates are clustered together as a sister group of MASP1/3s from Vertebrates, suggesting that C1rs and MASP1/2/3s in Vertebrates are closely related. MASPs from Cephalochordata, Urochordata, Hemichordata, and Echinodermata are clustered together and they are close to Vertebrate MASP2s and C1ss, suggesting that there is a close relationship among MASPs, C1rs, and C1ss in Deuterostomata. MASPLs from Arthropoda, Mollusca, and Annelida are clustered together and are close to C1ss from Vertebrates. These results also indicate that MASPLs from lower invertebrates may also be homologs of C1ss, suggesting that MASPLs from lower invertebrates may be the early prototype of Vertebrate C1ss, C1rs, and MASPs.

C2, BF, and DF are the crucial proteases in the classical pathway, lectin pathway, and alternative pathway [1,10,29]. Among them, C2 operates in both the classical and lectin pathways. BF and DF as the upstream and downstream proteases operate the alternative pathway. In the present study, C2 and BF both are traced to Coelenterata, they are lost in some phyla. Similarly, they are present in some species and found to be lost in others in the same phyla. The structural domains of C2s and BFs are conserved, and they all have the CCP_1–7_-VWA-Tyrp_SPc_1–2_ domain. The evolutionary tree was constructed to further analyze their relationship. No clear C2 branch or BF branch is observed in the evolutionary tree. The results indicate that C2 and BF are closely related to each other in the same phyla, suggesting that they may have co-evolved. DF with only one classical Tyrp_SPc domain is widely present in different metazoan phyla from Porifera to Chordata, suggesting that it is evolutionarily conserved. And the presence of DF in Porifera suggests that the primitive alternative pathway may be traced to Porifera.

C3, C4, and C5 are the crucial multi-chain proteins in the complement system. Among them, C3 as the central component in the complement system. C4 in the classical and lectin pathways is cleaved by C1s and the activated C4b binds to C2 to form C3 convertase (C4b2a), leading to C3 cleavage [39]. The activated C3b forms a covalent bond with C3b or C4b of C3 convertases (C3bBb and C4bC2a), switching their specificity to C5 convertases. The activated C5 initiates assembly of late components, C6 to C9, leading to the formation of MACs [1,2,40]. Evidence indicates that the appearance of a C3-like protein occurred at least a billion years ago [2]. In the present study, C3 can be traced back to Coelenterata. C4 and C5 were only present in Vertebrates. Most C3s all have the classical A2M_N-A2M_N_2-A2M-Thiol-A2M_comp-A2MR-C345C domain, suggesting that C3 is an evolutionarily conservative molecule. C4s and C5s from Vertebrates all have the classical A2M_N-A2M_N_2-A2M-A2M_comp-A2MR-C345C domain. The evolutionary tree was constructed to further analyze their evolution relationship. There are clearly three branches (C3 branch, C4 branch, and C5 branch), suggesting that C4 and C5 evolved from C3, and they keep evolutionarily independent in the evolution of vertebrate species. The production of C4 and C5 from C3 may also be caused by replication of the fish genome [41,42].

The terminal complement components (C6, C7, C8, and C9) and C5b assemble to form MAC, which forms pores on the plasma membrane of the target cell, disturbs the membrane potential, and finally leads to cell lysis [2,40]. Until now, the classical MAC can be traced back to Chondrichthyes with the presence of C6–C9. In addition, in lamprey, although the MAC components were lost, a primitive MAC was found to be composed of lamprey pore-forming protein (LPFP) [43]. In the present study, to trace the origins and evolution of MAC, the main components, such as C6, C7, C8, and C9, were screened from the well-annotated genomes of species in different subphyla/phyla. They are present in Vertebrates (except for Cyclostomata). In invertebrates, C6L and C7L are also in Cephalochordata, and C6L and C8 are in Urochordata. These results also reveal that the original MAC may be in Ascidian and Amphioxus. Most C6s and C7s in Vertebrates have the classical TSP1-TSP1-LDLa-MACPF-EGF_0–1_-TSP1-CCP_1–2_-FIMAC-FIMAC domain. Meanwhile, C6Ls in Cephalochordata and Urochordata have the TSP1-TSP1-LDLa-MACPF-EGF-TSP1_0–1_ domain, lacking the FIMAC domain. C7L from Cephalochordata only has the EGF-TSP1 domain or TSP1-FIMAC domain. C8s and C9s in Vertebrates all have the classical TSP1_0–1_-LDLa-MACPF-EGF_0–1_-TSP1_0–1_ domain. In addition, C8 from Urochordata has the LDLa-MACPF domain. In Vertebrates, C6s, C7s, C8s, and C9s are relatively conserved in evolution. A comprehensive analysis of the domain architectures of MAC components was conducted to investigate the origin of MAC. C6 from Amphioxus evolves from C6 from Ascidian. C6 and C8 from Ascidian and C7 from Amphioxus provide the structural conditions for Vertebrate MAC components. Among them, C6 and C8 offer the TSP1, MACPF, and EGF domains, and C7 provides the EGF, TSP1, and primitive FIMAC domains. These structural foundations along with replication of the fish genome eventually form C6, C7, C8, and C9 containing classical TSP1, MACPF, EGF, and/or FIMAC. However, these molecules are lost in Cyclostomata, which also suggests that there are large changes at the genome level during the evolution from invertebrates to vertebrates, leading to the loss of some genes and the presence of some genes. The above results collectively support the point that C4 and C5 were most probably generated by gene duplication from C3, C2 was generated from Bf, C1r/C1s from MASP, and C9/C7 from C6/C8, which eventually establish the complete complement system [41,42,44].

**Figure 11:**
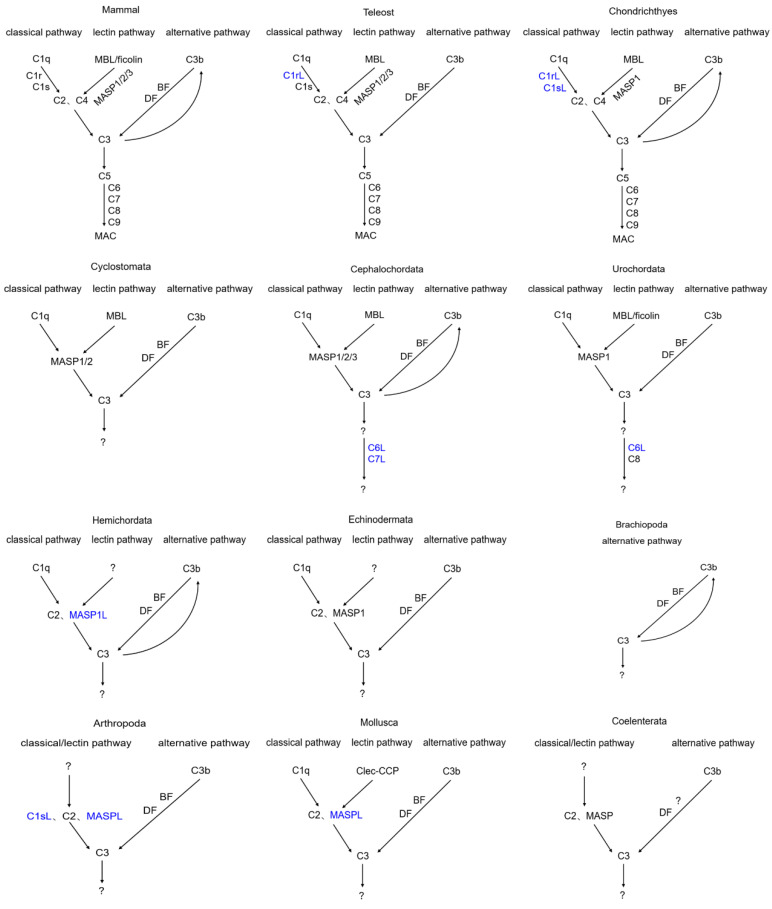
Activation of the complement system in different metazoan subphyla/phyla. The evolution of the complement system is accompanied by the appearance or loss of some genes and their domain rearrangement and/or reshuffling. Complement-like components are marked with blue. The question mark represented the corresponding gene not found in the phylum.

The complement systems of different metazoan subphyla/phyla are preliminarily outlined in Figure 11. There are three complement activation pathways in mammals, including the classical pathway, lectin pathway, and alternative pathway [1,11]. Activation of the complement system can promote the formation of MAC. The complete classical pathway can be traced back to Chondrichthyes. In Cyclostomata, there was one report demonstrated that lamprey C1q directly copurified with MASP-A to exhibit proteolytic activity against lamprey C3 [45]. And in mammals, C3 also can be directly activated by MASPs. So, it can be speculated that, in lower species with the absence of C1r, C1s, C2, and C4, MASP can replace them to directly bind to C1q/MBL and then be activated, ultimately promoting the cleavage of C3. Ficolin with a collagen-like domain is lacking in Teleosts and even lower phyla (except for Urochordata), so whether ficolin lacking a collagen-like domain can activate the lectin pathway still needs to be further investigated. Meanwhile, ficolin with a potential collagen-like domain and Clec-CCP with a CCP domain [37] are found in Urochordata and Mollusca, respectively, and Clec-CCP from oyster is demonstrated to be able to activate the MASPL-C3-mediated lectin pathway [37]. In the absence of the classical C1q, MBL, and ficolin, the activation mechanism of the alternative pathway as well as C3 still needs to be further investigated in Brachiopoda, Arthropoda, and Coelenterata. In MAC, the main components are C5/C6/C7/C8/C9, which are in Mammals, Teleosts, and Chondrichthyes, suggesting the presence of MAC. In Cyclostomata, C5, C6, C7, C8, and C9 are not all present. Meanwhile, C6L and C7L are found in Cephalochordata, and C6L and C8 are in Urochordata, suggesting that MAC is evolved from Cephalochordata and Urochordata. The existence of MAC in Cephalochordata and Urochordata and its activation mechanism still need further confirmation.

**Figure 12:**
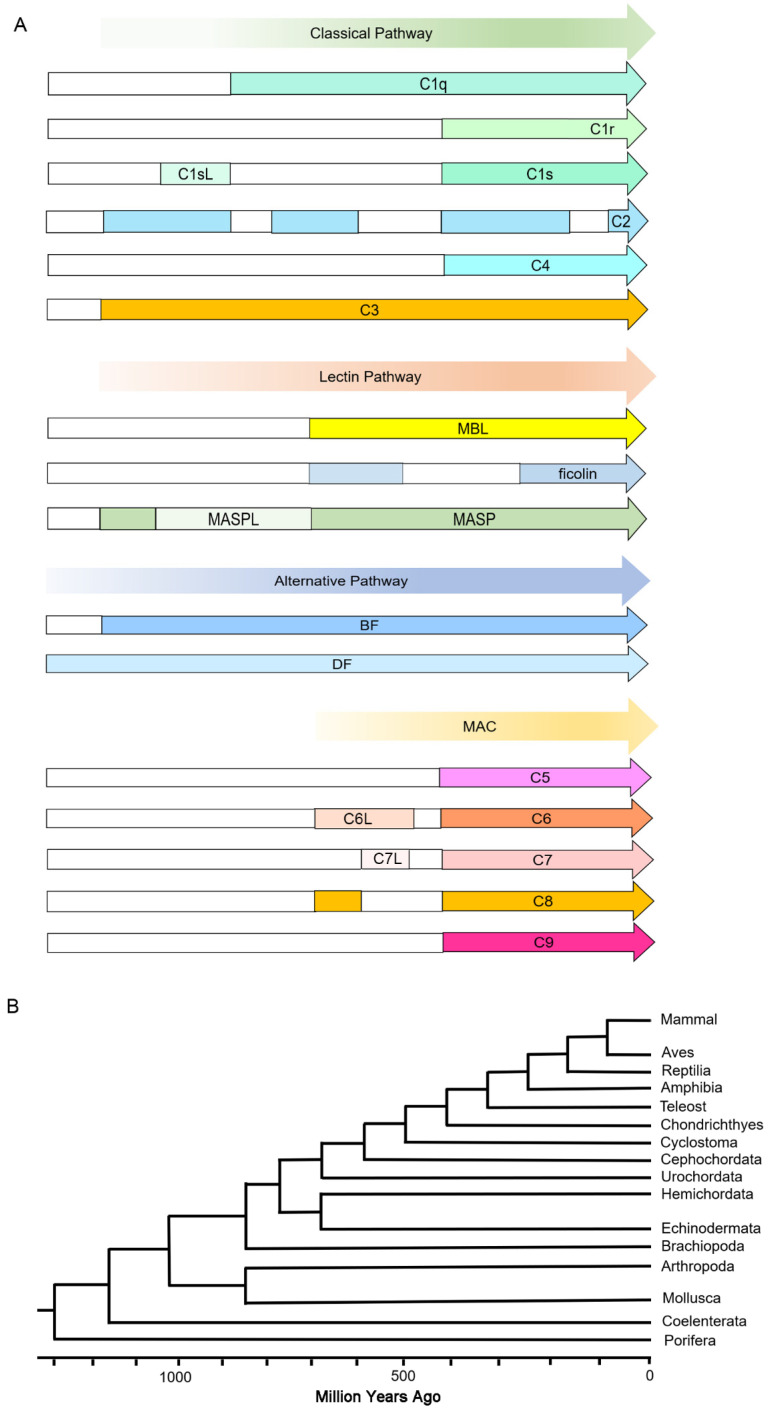
The evolutionary processes of the complement system. (**A**) Evolutionary origins of the three complement activation pathways shown by the colored arrows. The origin and evolution of the gene families of the complement system shown by the colored arrows with boxes. The white box indicates no gene exists. (**B**) Phylogenetic relationships among metazoan subphyla/phyla.

The current view of the complement system’s evolutionary processes is summarized in Figure 12. According to the evolution of metazoans and the presence of components in the complement system, it can be speculated that the classical pathway and lectin pathway first appeared at the same time in Coelenterata and the primitive alternative pathway first appeared in Porifera. So, the alternative pathway is the earliest of the three pathways in metazoans. With the presence of C6L, C7L, and C8 in Cephalochordata and Urochordata, MAC may have originated from Cephalochordata and Urochordata. And the complete components (C5/C6/C7/C8/C9) in MAC first appeared in Chondrichthyes. Taken together, the earliest pathway is the alternative pathway, followed by the lectin and classical pathways, and the terminal pathway can be traced back to Cephalochordata and Urochordata. The findings help us to better understand the evolutionary principles of the complement system and its function, which will be employed to control and redesign the pathway for potential applications.

## 4. Materials and Methods

### 4.1. The Amino Acid Sequences of Complement Components

The amino acid sequences of the complement components were obtained by screening the genomes of different species in metazoan subphyla/phyla from the National Center for Biotechnology Information (NCBI) database (https://www.ncbi.nlm.nih.gov/ (accessed on 15 July 2024)). The classical complement components from different species in metazoan subphyla/phyla were directly download from the NCBI database and the screened amino acid sequences were analyzed using Research Tool (SMART) (http://www.smart.embl-heidelberg.de/ (accessed on 15 July 2024)) to compare the differences in their structural domains, which eventually obtained the required sequences. Blastp (https://blast.ncbi.nlm.nih.gov/Blast.cgi (accessed on 15 July 2024)) was used to screen the nonclassical complement components by using the identified complement components from species. The obtained sequences were then analyzed as mentioned above. The complement components include C1q, C1r, C1s, C2, C4, C3, ficolin, MBL, MASP, C5, C6, C7, C8, and C9. The species are as follows: *Homo sapiens*, *Mus musculus*, *Motacilla alba alba*, *Xenopus laevis*, *Bufo bufo*, *Chelonia mydas*, *Oncorhynchus mykiss*, *Danio rerio*, *Latimeria chalumnae*, *Anguilla anguilla*, *Hypanus sabinus*, *Petromyzon marinus*, *Ciona intestinalis, Stegostoma tigrinum*, *Branchiostoma belcheri*, *B. japonicum*, *B. floridae*, *Saccoglossus kowalevskii*, *Apostichopus japonicus*, *Strongylocentrotus purpuratus*, *Acanthaster planci*, *Lingula anatine*, *Owenia fusiformis*, *Arenicola marina*, *Crassostrea gigas*, *C. virginica*, *C. angulate*, *Ostrea edulis*, *Mercenaria mercenaria*, *Dreissena polymorpha*, *Ruditapes philippinarum*, *Mizuhopecten yessoensis*, *Sinonovacula constricta*, *Haliotis rufescens*, *Euprymna scolopes*, *Stegodyphus dumicola*, *Parasteatoda tepidariorum*, *Tropilaelaps mercedesae*, *Penaeus vannamei*, *Stegodyphus dumicola*, *Hasarius adansoni*, *Argiope bruennichi*, *Lineus ruber*, *Caenorhabditis elegans*, *Bugula neritina*, *Stylophora pistillata*, *Nematostella vectensis*, and *Amphimedon queenslandica*.

### 4.2. BLASTp Analysis of Complement Components

BLASTp was used to search the homologs of complement components in the genomes of different species. The obtained amino acid sequences were then analyzed using Research Tool (SMART) (http://www.smart.embl-heidelberg.de/ (accessed on 15 July 2024)) to further confirm the structural domain composition. The structural domains were compared with those of the complement components in mammals to identify the best complement components from genomes of different species.

### 4.3. The Evolutionary Analysis of Complement Components

In order to further trace the evolutionary history of different complement components, phylogenetic trees based on their full-length amino acid sequences were constructed using MEGA 11 software with the neighbor-joining (NJ) method. Bootstrapping with 1000 replications was conducted to evaluate the phylogenetic trees.

### 4.4. The Structural Domain Analysis of Complement Components

The structural domains of complement components from the major metazoan phyla were predicted by SMART. The structural domains of complement components were outlined using Microsoft Office PowerPoint (PPT). Conserved structural domains of complement components were confirmed by the NCBI (Bethesda, MD, USA) Conserved Domains Database.

### 4.5. The Evolutionary Tree of Metazoan

The evolutionary tree of metazoans was constructed according to the evolution of metazoans with time. They were Mammals, Aves, Reptilia, Amphibia, Teleosts, Chondrichthyes, Cyclostomata, Cephochordata, Urochordata, Hemichordata, Echinodermata, Brachiopoda, Arthropoda, Mollusca, Coelenterata, and Porifera.

## Figures and Tables

**Table 1:** The abbreviations for the domains used in the study.

Domain Abbreviation	Complete Name	Domain Abbreviation	Complete Name
Collagen	Collagen domain	CRD	Carbohydrate-recognition domain
C1q	C1q domain	CUB	C1r/C1s-Uegf-BMP domain
FREP	Fibrinogen-related domain	EGF	Epidermal growth factor
CCP	Complement control protein	Tryp_SPc	Trypsin-like serine protease
IG	Immunoglobulin	LDLa	Low density lipoprotein A
VWA	Von willebrand factor	A2M	Alpha-2-macroglobulin region
ANATO	Anaphylatoxin homologous domain	Thiol	Alpha-macro-globulin thiol-ester bond-forming region
A2M recep	Receptor domain region of the alpha-2-macroglobulin family	C345C	Netrin C-terminal Domain
TSP1	Thrombospondin type 1	MACPF	Membrane attack complex/perforin
FIMAC	Factor I membrane attack complex		

## Data Availability

No new data were generated or analyzed in support of this research.
